# Improved oxidation behavior of Hf_0.11_Al_0.20_B_0.69_ in comparison to Hf_0.28_B_0.72_ magnetron sputtered thin films

**DOI:** 10.1038/s41598-024-72134-3

**Published:** 2024-09-17

**Authors:** Pauline Kümmerl, Sebastian Lellig, Amir Hossein Navidi Kashani, Marcus Hans, Peter J. Pöllmann, Lukas Löfler, Ganesh Kumar Nayak, Damian M. Holzapfel, Szilárd Kolozsvári, Peter Polcik, Peter Schweizer, Daniel Primetzhofer, Johann Michler, Jochen M. Schneider

**Affiliations:** 1 Materials Chemistry, RWTH Aachen University, Kopernikusstr. 10, 52074 Aachen, Germany; 2 Empa, Swiss Federal Laboratories for Materials Science and Technology, Laboratory for Mechanics of Materials and Nanostructures, Feuerwerkerstrasse 39, 3602 Thun, Switzerland; 3 Plansee Composite Materials GmbH, Siebenbürgerstr. 23, 86963 Lechbruck am See, Germany; 4 Department of Physics and Astronomy, Uppsala University, Lägerhyddsvägen 1, 75120 Uppsala, Sweden

**Keywords:** Thin films, Coatings, Borides, High temperature oxidation, HfAlB_2_, Magnetron sputtering, Surfaces, interfaces and thin films, Design, synthesis and processing, Corrosion

## Abstract

The oxidation resistance of Hf_0.28_B_0.72_ and Hf_0.11_Al_0.20_B_0.69_ thin films was investigated comparatively at 700 °C for up to 8 h. Single-phase solid solution thin films were co-sputtered from HfB_2_ and AlB_2_ compound targets. After oxidation at 700 °C for 8 h an oxide scale thickness of 31 $\pm$ 2 nm was formed on Hf_0.11_Al_0.20_B_0.69_ which corresponds to 14% of the scale thickness measured on Hf_0.28_B_0.72_. The improved oxidation resistance can be rationalized based on the chemical composition and the morphology of the formed oxide scales. On Hf_0.28_B_0.72_ the formation of a porous, O, Hf, and B-containing scale and the formation of crystalline HfO_2_ is observed. Whereas on Hf_0.11_Al_0.20_B_0.69_ a dense, primarily amorphous scale containing O, Al, B as well as approximately 3 at% of Hf forms, which reduces the oxidation kinetics significantly by passivation. Benchmarking Hf_0.11_Al_0.20_B_0.69_ with Ti–Al-based boride and nitride thin films with similar Al concentrations reveals superior oxidation behavior of the Hf-Al-based thin film. The incorporation of few at% of Hf in the oxide scale decelerates oxidation kinetics at 700 °C and leads to a reduction in oxide scale thickness of 21% and 47% compared to Ti_0.12_Al_0.21_B_0.67_ and Ti_0.27_Al_0.21_N_0.52_, respectively. Contrary to Ti–Al-based diborides, Hf_0.11_Al_0.20_B_0.69_ shows excellent oxidation behavior despite B-richness.

## Introduction

Transition metal diborides (TMB_2_) comprise a combination of covalent-ionic and metallic bonding between B–B, B–TM, and TM–TM^1,2^. Therefore, TMB_2_ possess a variety of desirable ceramic-like material properties, such as high hardness^1,3^ and elastic modulus^1,4,5^, good chemical stability^6,7^ and corrosion resistance^8^, as well as properties related to the metallic bonding character like high electrical^1^ and thermal conductivity^1,4^, and relatively large thermal shock resistance compared to other ceramics^9^. The highest oxidation resistance among TMB_2_ is reported for HfB_2_^10^, which belongs with a melting point of 3250 °C^11^, to the so-called ultra-high temperature ceramics (UHTC)^1^. Previous research on HfB_2_-based materials has therefore focused on high-temperature applications for the aerospace industry, mainly on the HfB_2_-SiC system, to increase the heat resistance of structural thermal protection systems to be used as heat shields for re-entry vehicles^9,12,13^.

HfB_2_ crystallizes—as most TMB_2_—in the hexagonal AlB_2_ type crystal structure^14^ in the space group P6/mmm with lattice constants *a* $=$ 3.142 Å and *c* $=$ 3.476 Å^15^. The AlB_2_-type crystal structure is characterized by hexagonal closed-packed layers of TM atoms, with graphite-like atomic sheets of B interspersed between them. This structural arrangement gives rise to the above-discussed combination of covalent-ionic and metallic bonding characters^4,16,17^.

Gild et al.^10^ compared the oxidation resistance of six different bulk high-entropy transition metal diborides, namely, (Hf_0.2_Zr_0.2_Ta_0.2_Nb_0.2_Ti_0.2_)B_2_, (Hf_0.2_Zr_0.2_Ta_0.2_Mo_0.2_Ti_0.2_)B_2_, (Hf_0.2_Zr_0.2_Mo_0.2_Nb_0.2_Ti_0.2_)B_2_, (Hf_0.2_Mo_0.2_Ta_0.2_Nb_0.2_Ti_0.2_)B_2_, (Mo_0.2_Zr_0.2_Ta_0.2_Nb_0.2_Ti_0.2_)B_2_, and (Hf_0.2_Zr_0.2_Ta_0.2_Cr_0.2_Ti_0.2_)B_2_, to their corresponding, binary transition metal diborides. Generally, the high-entropy transition metal diborides outperformed the individual binary TMB_2_^10^. However, HfB_2_ was reported to exhibit a very similar weight gain after oxidation for 1 h at 1000 °C, 1100 °C, and 1200 °C compared to the best-performing high-entropy metal diborides, namely (Hf_0.2_Zr_0.2_Ta_0.2_Mo_0.2_Ti_0.2_)B_2_, (Hf_0.2_Mo_0.2_Ta_0.2_Nb_0.2_Ti_0.2_)B_2_ and (Hf_0.2_Zr_0.2_Ta_0.2_Cr_0.2_Ti_0.2_)B_2_. At 1500 °C HfB_2_ outperformed all high-entropy metal diborides underlining its high oxidation resistance^10^.

HfB_2_ coatings have first been synthesized by chemical vapor deposition and have been studied in terms of wear resistance for tribological applications^18–22^. However, recently chemical composition quantification^23^ and oxidation behavior^24^ of direct current magnetron sputtered (DCMS) HfB_2_ coatings have been reported. Glechner et al.^24^ performed oxidation experiments on Hf_0.30_B_0.70_ in synthetic air with significantly lower water vapor pressure than in ambient air. An increase in mass during dynamic oxidation experiments was observed up to a temperature of 1200 °C, followed by a decrease in mass. This behavior was explained by the evaporation of B_2_O_3_ above 1200 °C. After 120 min of oxidation at 700 °C, a crystalline oxide containing O, Hf, and B with a scale thickness of 171 $\pm$ 21 nm was observed. After oxidation at 800 °C and 900 °C, an additional porous B-rich layer formed on top of a Hf-rich oxide containing O, Hf, and B^24^. However, when oxidation was carried out in ambient air within the same report, no boron oxide was observed which was attributed to the hygroscopic character of B_2_O_3_ forming volatile compounds^5,24^. Based on secondary ion mass spectrometry (SIMS) data the authors concluded that Hf_0.30_B_0.70_ films show very limited oxygen inward diffusion (through the scale) and that these films are hence, efficient oxygen diffusion barriers^24^.

For bulk samples, it was shown that the oxidation resistance of ZrB_2_ and HfB_2_ can be improved by the addition of SiC^25–27^. For coatings, it has been demonstrated that the addition of Si into TMB_2_ (TM = Ti, Cr, Hf, Ta, W)^28,29^ or Al^30^ into TiB_2_ leads to enhanced oxidation resistance: For Ti_0.29_Al_0.13_B_0.58_ a protective Al-containing scale forms during oxidation at 700 °C for up to 8 h which reduced the oxide scale thickness from ~ 1350 nm for the Ti_0.29_B_0.71_ coating to ~ 460 nm for the Ti_0.29_Al_0.13_B_0.58_ coating^30^.

Furthermore, the chemical composition and especially the boron-to-metal ratio play a very important role in the oxidation resistance of TMB_2_. Under-stoichiometric Ti_0.41_B_0.59_ (boron-to-metal ratio = 1.4) films have been reported to exhibit superior oxidation resistance compared to over-stoichiometric Ti_0.32_B_0.68_ and Ti_0.27_B_0.73_ (boron-to-metal ratio of 2.2 and 2.7 respectively) films due to the absence of the B-tissue phase, resulting in the formation of a denser TiO_x_ oxide scale with reduced B_2_O_3_ evaporation, thereby limiting oxide scale growth^31^.

One method to deposit stoichiometric Ti–Al–B coatings was shown by Stüber et al.^32^ to co-sputter elemental Al to TiB_2_ forming a Ti_0.84_Al_0.16_B_2.0_ solid solution. Another deposition approach was chosen by Navidi Kashani et al.^33^ who studied the effect of the B concentration on the mechanical properties and the oxidation resistance at 700 °C for up to 8 h for Ti–Al–B coatings. Using elemental B targets together with a TiAl target, Ti–Al–B coatings with an Al concentration range of 22 ± 2 at% and varying B concentration from 63 via 67 to 71 at% and hence, above and below stoichiometry, were synthesized. The most favorable combination of the oxidation resistance and mechanical behavior was obtained for the stoichiometric Ti_0.12_Al_0.21_B_0.67_ coating with an oxide scale thickness of 39 $\pm$ 7 nm. In contrast, the over-stoichiometric Ti_0.10_Al_0.19_B_0.71_ coating had a factor 5.2 thicker oxide scale of 204 $\pm$ 16 nm after 8 h^33^. Recently, oxidation experiments in ambient air at 700 °C, 800 °C, and 900 °C revealed a strong Al concentration dependence of the oxidation resistance. Stoichiometric films containing ≥ 21 at% of Al formed a dense and passivating oxide scale, while Al concentrations ≤ 15 at% resulted in the formation of a non-passivating scale^34^.

Here, we seek to explore, if the oxidation resistance of Hf_0.28_B_0.72_ thin films can be further improved by Al incorporation. An oxidation temperature of 700 °C was chosen to study the onset of oxidation and compare it with literature data for potential coating materials. To this end, the oxidation resistance and oxide scale formation of Hf_0.11_Al_0.20_B_0.69_ is compared to Hf_0.28_B_0.72_ as well as to the available literature data for Hf-B, Ti–Al–B, and Ti–Al–N films exhibiting similar Al concentrations.

## Results and discussion

Sputtering only the HfB_2_ target leads to a film with 27.5 $\pm$ 3.5 at% Hf, 72.2 $\pm$ 3.6 at% B, and 0.3 $\pm$ 0.1 at% O according to elastic recoil detection analysis (ToF-ERDA) and will be referred to as Hf_0.28_B_0.72_ in the following discussion. The B-to-Hf ratio of 2.6 indicates that the film is B over-stoichiometric compared to stoichiometric HfB_2_.

This B-richness is in good agreement with other literature reports for DCMS-deposited HfB_*x*_ thin films from stoichiometric HfB_2_ compound targets^24,35^. The deviation of the film composition from the target composition for constituents with large differences in mass has been proposed to be caused by preferential resputtering of one of the sputtered constituents^36–38^ and/or preferential sputtering of the lighter constituent along the target normal^39^. For TiB_*x*_ thin films, Neidhardt et al.^40^ have demonstrated experimentally and with simulations that there is a complex interaction between the angular distribution of the sputtered flux as well as gas scattering which influences the film composition. The preferential emission of B along the target normal is observed while the angular distribution of Ti is much broader and similar to the ideal spherical cosine distribution. These emission characteristics are modified at higher pressures by the effect of gas scattering. Because of the larger atomic radius, Ti has a more efficient energy transfer with Ar and a shorter thermal mean free path (MFP) than B. This results in more effective stopping and thus less scattering of Ti than B. The complex interdependence between emission characteristics and scattering processescan explain the linear dependence of the Ti/B ratio with pressure and the substrate-to-target distance. As a result, at low pressure, no scattering collisions are expected for B leading to a Ti-deficient film. At high pressures, however, B is scattered preferentially, resulting in a B-deficient film^40^. Mráz et al.^41^ studied the angle-resolved thin film composition evolution during sputtering of a Cr_2_AlC compound target by varying the angle $\alpha$ between the target normal and the substrate surface between 0° and 67.5°. As the mass differences in this system are larger than for TiB_2_, significant differences between film and target composition were reported: for *α* ≤ 22.5°heavy element deficient thin films compared to the target composition are observed where the deficiency decreases with increasing pressure. However, this heavy element deficiency is decreasing with increasing angle between the target normal and the substrate surface^41^.

In Hf_0.28_B_0.72_ the mass difference between the target constituents is larger and $\alpha$ $=$ 0°. Hence, the angular distribution of Hf and B differs more severely than in TiB_2_^40^ and the formation of Hf-deficient thin films is expected based on the reasoning in^40^ and^41^.

To estimate the effect of gas phase scattering the MFP was calculated using Eq. (1) which includes the deposition temperature (*T* = 673 K), deposition pressure (*p* = 0.6 Pa), covalent radii of Ar (*r*_*Ar*_ = 71 pm^42^), Hf (*r*_*Hf*_ = 208 pm^42^) or B (*r*_*B*_ = 87 pm^42^).

1 $$MFP=\frac{1}{\sqrt{2}}\frac{{k}_{B}T}{p}\frac{1}{\pi {({r}_{Ar}+{r}_{Hf,B})}^{2}}$$

The MFP of B is 14.0 cm leading at a substrate-to-target distance of 10 cm to an average number of 0.7 collisions. An average of < 1 collision implies that B atoms will likely not undergo collisions on route to the target and will therefore likely be incorporated in the growing thin film. In contrast, the MFP of Hf is 4.5 cm resulting in 2.2 collisions on average, causing less efficient transport of Hf to the surface of the growing film which is in turn leading to a Hf-deficient and hence B-rich thin film^40^. Therefore, the here observed Hf deficiency (compared to the target composition) can be attributed to preferential emission of B along the target normal as well as efficient scattering of Hf.

When AlB_2_ is co-sputtered with HfB_2_ at the same angle $\alpha$ between the target normal and the substrate surface, the B over-stoichiometry decreases to a B/(Hf + Al) ratio of 2.3 which corresponds to 69.2 $\pm$ 3.5 at% B and Al and Hf concentrations of 19.8 $\pm$ 2.2 at% and 10.5 $\pm$ 1.2 at%, respectively. Henceforth, the film will be referred to as Hf_0.11_Al_0.20_B_0.69_. The oxygen concentration was 0.5 $\pm$ 0.1 at%. The reduced B over-stoichiometry compared to Hf_0.28_B_0.72_ can be explained by the smaller mass (*m*_*Al*_ = 26.98 u) and atomic radius (*r*_*Al*_ = 118 pm^42^) of Al compared to Hf with *m*_*Hf*_ = 178.49 u and *r*_*Hf*_ = 208 pm^42^, which leads to an MFP of 9.8 cm and therefore to an average number of 1.0 collisions and thus to more efficient Al transport towards the surface of the growing film. Hence, as more metal is incorporated into the film the B-richness is reduced.

**Fig. 1:**
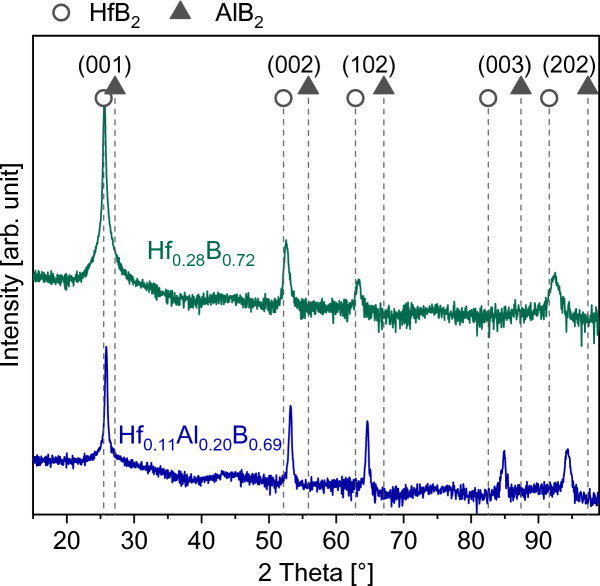
Diffraction patterns of as-deposited Hf_0.28_B_0.72_ and Hf_0.11_Al_0.20_B_0.69_ thin films.

Figure 1 depicts X-ray diffraction (XRD) patterns of the as-deposited films grown on α-Al_2_O_3_ (0001) to investigate the phase formation. The positions of HfB_2_ and AlB_2_ reference lines according to the JCPDS cards 00-038-1398 and 00-008-0216 are highlighted with circle and triangle markers, respectively. The diffractogram of Hf_0.28_B_0.72_ reveals the formation of a single-phase hexagonal HfB_2_ structure. The peak positions of the Hf_0.11_Al_0.20_B_0.69_ films are located between those of the binary HfB_2_ and AlB_2_ phases, indicating the formation of a solid solution. Based on our DFT data this solid solution is metastable as witnessed by the positive mixing enthalpy of 0.08 eV/atom for stoichiometric Hf_0.12_Al_0.21_B_0.67_.

**Fig. 2:**
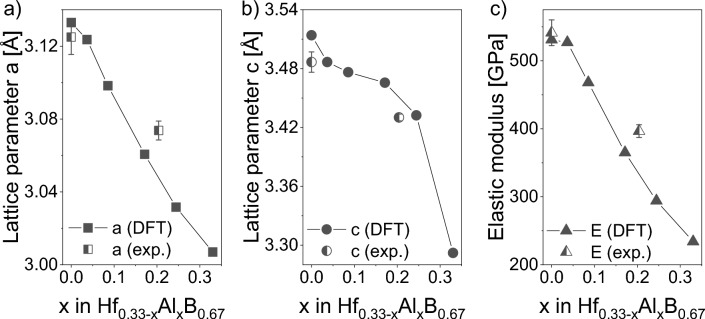
Change in lattice parameters (**a**) a and (**b**) c as well as (**c**) the elastic modulus as a function of the Al concentration x in Hf_0.33−x_Al_x_B_0.67_ calculated by DFT and compared with experimental data.

Figure 2 shows the calculated and experimentally obtained lattice constants *a* and *c* as a function of Al concentration *x* in Hf_0.33−x_Al_x_B_0.67._ While the calculated lattice constant *a* decreases almost linearly with the Al concentration following Vegard´s law^43^, the *c* parameter exhibits a non-linear behavior. The deviation of the experimental to the density functional theory (DFT) calculated lattice parameter of Hf_0.28_B_0.72_ is − 0.3% for *a* and − 0.8% for *c* and of Hf_0.11_Al_0.20_B_0.69_ it is + 0.9% for *a* and − 0.6% for *c*, respectively. According to Paier et al., deviations of up to 2.1% between DFT-calculated and experimental lattice parameters have to be expected based on the here-applied exchange–correlation functionals^44^. Hence, very good agreement between prediction and experiment is obtained even though the B over-stoichiometry and the actual stress state of the as-deposited thin films were not considered.

**Fig. 3:**
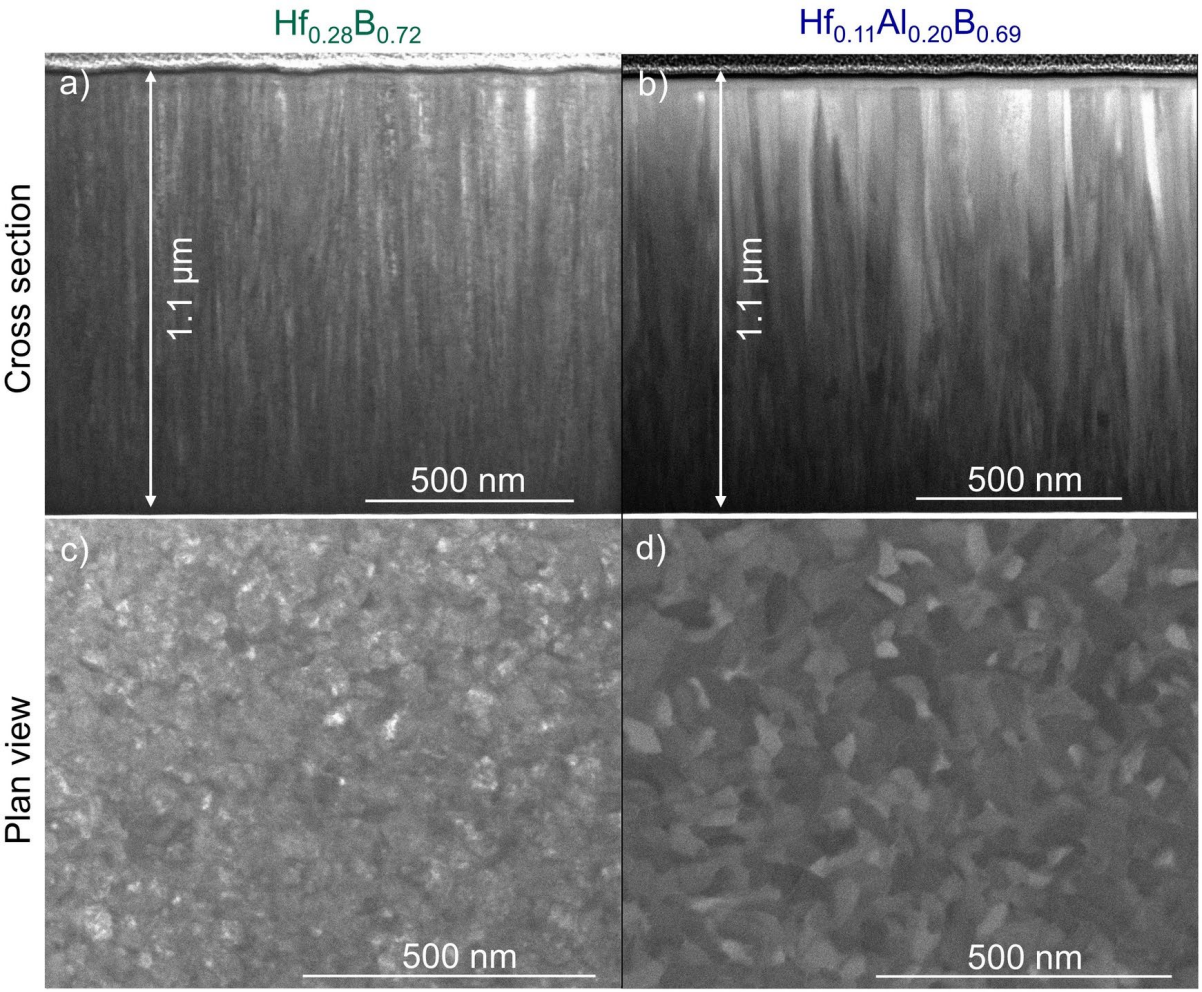
STEM images of as-deposited Hf_0.28_B_0.72_ (**a** and **c**) and Hf_0.11_Al_0.20_B_0.69_ (**b** and **d**) thin films in BF-mode from cross-sectional (**a** and **b**) and plan view (c and d).

To study the morphology, cross-sectional and plan view scanning transmission electron microscopy (STEM) images in bright-field (BF) mode were taken which are shown in Fig. 3. Hf_0.28_B_0.72_ exhibits a small-grained, dense, and nanocolumnar microstructure. While this observation is consistent with literature reports from Mayrhofer et al.^3^ where the excess boron is reported to surround the columns and between smaller sub-columns, the formation of a B-rich tissue phase cannot be inferred from the images presented here.

The ternary solid solution film also shows a dense, columnar morphology; however, the column size is increased as depicted in Fig. 3b,d compared to Fig. 3a,c. The hardness and elastic modulus determined for the Hf_0.28_B_0.72_ film are 38 $\pm$ 2 GPa and 541 $\pm$ 19 GPa, respectively, which is reduced to 30 $\pm$ 1 GPa and 397 $\pm$ 9 GPa after Al addition forming Hf_0.11_Al_0.20_B_0.69._ As depicted in Fig. 2, the elastic modulus of Hf_0.28_B_0.72_ matches the values predicted by DFT within the error bar. The experimentally determined elastic modulus of Hf_0.11_Al_0.20_B_0.69_ is 18.5% higher than the DFT calculated value of 334 GPa, which is according to Paier et al.^44^ within the expected range of bulk modulus deviations between different exchange–correlation functionals and experiments. As the Al concentration is increased from 0 at% via 20 at% to 33 at% in Hf_0.33*−x*_Al_*x*_B_0.67_, the cohesive energy increases linearly from − 7.87 eV/atom via − 6.44 eV/atom to − 5.68 eV/atom, respectively. The increase in cohesive energy indicates that the reduction in elastic modulus can be explained by bond weakening caused by Al addition as observed in Ti_0.33−*x*_Al_*x*_B_0.67_^34^. This bond weakening leads to an elastic modulus reduction of ~ 7 GPa per at% of Al in Hf_0.33−*x*_Al_*x*_B_0.67._ Al concentration induced bond weakening has also been reported for Ti_0.33−*x*_Al_*x*_B_0.67_, where also a ~ 7 GPa reduction was observed for each at% Al added^34^.

**Fig. 4:**
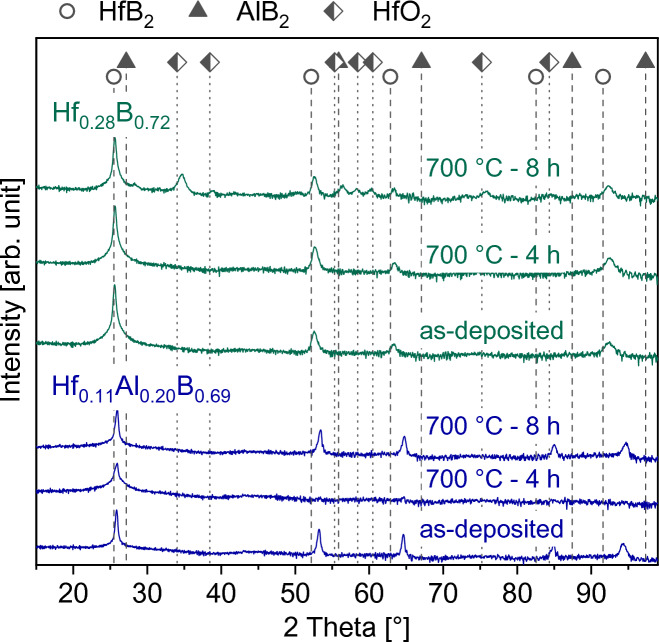
Diffraction patterns of as-deposited and oxidized Hf_0.28_B_0.72_ and Hf_0.11_Al_0.20_B_0.69_ thin films at 700 °C.

The time-dependent oxidation resistance at 700 °C of the ternary solid solution Hf_0.11_Al_0.20_B_0.69_ thin film was compared to the binary Hf_0.28_B_0.72_ thin film. In Fig. 4 the diffractograms of the as-deposited and oxidized films after 4 and 8 h are presented. The positions of HfO_2_ reference lines according to the JCPDS card 01-070-2832 is shown by the square marker in addition to the positions of AlB_2_ and HfB_2_ from Fig. 1. It is evident that for Hf_0.28_B_0.72_ crystalline HfO_2_ is formed between 4 and 8 h of oxidation. In contrast, for Hf_0.11_Al_0.20_B_0.69_ no additional diffraction signals are visible, even after 8 h of oxidation, indicating the absence of X-ray crystalline oxide scales.

**Fig. 5:**
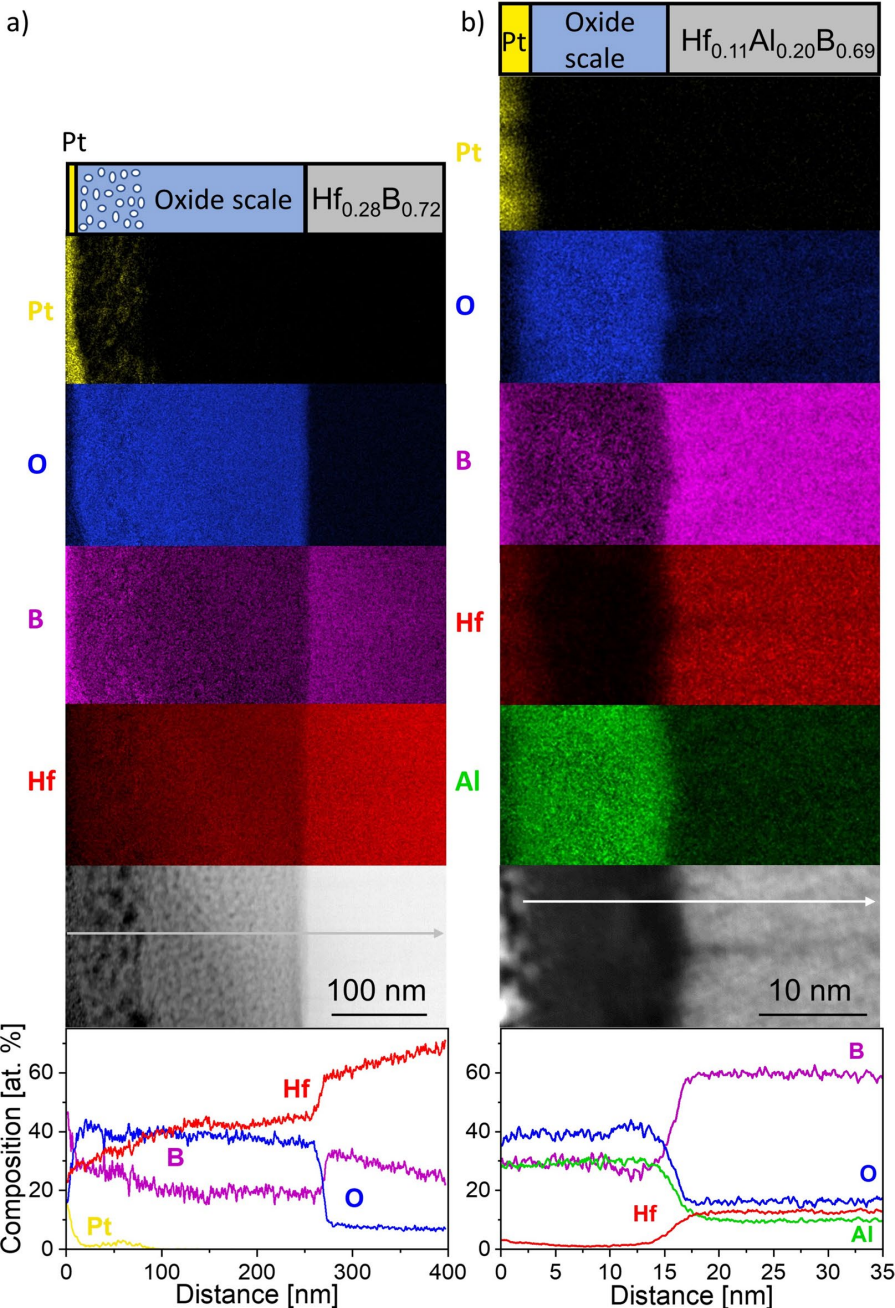
HRSTEM EDX line scans, EDX maps, and a HAADF cross-section image of the oxidized (**a**) Hf_0.28_B_0.72_ and (**b**) Hf_0.11_Al_0.20_B_0.69_ after 8 h at 700 °C, where the grey arrows mark the positions of the line scans.

Figure 5 shows a high angle annular dark field (HAADF) high-resolution STEM (HRSTEM) micrograph along with corresponding energy dispersive X-ray spectroscopy (EDX) maps and line scans capturing the oxide scale including the interfacial region as well as the underlying unaffected thin film for oxidized Hf_0.28_B_0.72_ and Hf_0.11_Al_0.20_B_0.69_ after 8 h at 700 °C. It should be noted that due to the difference in oxide scale thickness of Hf_0.28_B_0.72_ and Hf_0.09_Al_0.21_B_0.70_, the scale bars in the images and the corresponding EDX maps differ.

The HAADF image of Hf_0.28_B_0.72_, see Fig. 5a, shows a bi-layered oxide scale morphology, where the ~ 100 nm thick top layer is more porous than the ~ 160 nm thick layer below. The corresponding EDX line scan and maps indicate the formation of an O, Hf, and B-containing oxide. The B concentration gradually decreases while the Hf concentration increases from the oxide surface towards the film. The fact that Pt, utilized during lamellae preparation, was able to penetrate the oxide scale could amplify the decrease in the Hf concentration measured in the line scan. Furthermore, literature shows that B quantification in TEM–EDX spectra is strongly affected by the lamellae thickness due to preferential B-K_α_ X-ray absorption with increasing film thickness^45^. Hence, we assume that the increasing lamellae thickness towards the substrate is causing the above-discussed composition variations.

Similar to the present study, Glechner et al.^24^ reported a bi-layered oxide scale formation for Hf_0.30_B_0.70_ thin films oxidized in synthetic air, with a B-rich surface-near oxide layer and a Hf-rich oxide layer underneath based on SIMS data. However, the porous boron oxide layer was based on electron microscopy cross-sections only described for Hf_0.30_B_0.70_ thin films oxidized above 800 °C and the boron oxide on the surface only for synthetic air experiments. When the experiment was repeated in ambient air, no boron oxide was found on the surface which was explained by the formation of volatile H_m_BO_n_ in the presence of water vapor which evaporates earlier. However, there is no detailed description of the composition and morphology of the oxide scale at 700 °C for the oxidation experiment conducted in ambient air^24^.

In this study, after oxidation at 700 °C for 8 h, the formation of crystalline HfO_2_ is detected by XRD concurrent with EDX which indicates that the oxide scale consists of O, Hf, and B, while the HRSTEM image shows a porous morphology. Based on the oxidation model for bulk HfB_2_ from Parthasarathy et al.^46^, for oxidation temperatures < 1000 °C a glassy B_2_O_3_ film is expected to form on top of a porous, crystalline HfO_2_ scale, where the pores are filled with B_2_O_3_. At temperatures > 1000 °C B_2_O_3_ at the surface starts to evaporate^46^.

However, experiments and thermodynamic calculations with water-saturated synthetic air, with a water vapor pressure of 0.031 atm at 25 °C, showed that in the presence of water vapor B_2_O_3_ reacts and forms hydrated B–O compounds like HBO_2_ and H_3_BO_3_ which evaporate below 1000 °C^5,47^.

There are opposing reports in the literature about the rate of B_2_O_3,_ or more specifically H_m_BO_n_ evaporation, at ~ 700 °C in ambient air. Some authors report an almost complete absence of the B_2_O_3_ phase for Ti-B films oxidized in synthetic air with 40% humidity^47^ and Hf-B films oxidized in ambient air^24^. However, there are also reports of the presence of B_2_O_3_ in the oxide scale forming on TiB_2_ bulk samples oxidized in ambient air at this temperature^48^ or even at 1200 °C for 1 h of oxidation of ZrB_2_ bulk samples oxidized in water-saturated synthetic air^5^. This suggests that a Hf and B containing oxide scalesimilar to the oxidation model for bulk HfB_2_ has formed on the here investigated Hf_0.28_B_0.72_ where some B_2_O_3_ has started to form H_m_BO_n_ and thus has evaporated leaving pores in the oxide scale in the vicinity of the surface. However, also here the discussed limitation in B quantification for films with decreasing thickness applies^45^. Therefore, we conclude that based on the EDX and TEM results, a Hf-B containing oxide scale has formed where some B has formed volatile H_m_BO_n_ and evaporated leaving pores. Still, significant amounts of B are still present in the scale after oxidation at 700 °C for 8 h.

The oxide scale forming on Hf_0.11_Al_0.20_B_0.69_ is dense and contains besides O also equal amounts of Al and B as well as approximately 3 at% of Hf. The EDX maps show that the elements are homogeneously distributed. Based on ERDA measurements, the O concentration in the remaining film is below 1 at% and thus significantly lower than the ~ 18 at% O concentration measured by EDX. This observation can be explained by the sample preparation procedure. For the HRSTEM EDX analysis, a $\sim$ 60 nm thin lamella is prepared in the focused ion beam (FIB) and subsequently transferred through ambient air. Thus, it is reasonable to assume that oxidation by atmosphere exposure causes the formation of native oxides on each side of the lamella, which will affect the measured amount of O in EDX. Therefore, the Hf_0.28_B_0.72_ film exhibits ~ 10 at% of O. The 8 at% higher O content in Hf_0.11_Al_0.20_B_0.69_ might be related to the higher Al concentration in the film.

Moreover, EDX is not well positioned to quantify the boron content, since light elements have low characteristic X-ray energies and a low yield resulting in difficulties in accurate resolution of their signals against background noise and limitations in detector sensitivity^49^. As a consequence, the accuracy of boron concentration measurements obtained by EDX is limited, and previous studies have reported systematic overestimation of boron concentration in Hf-B thin films using this technique when compared to ERDA^23^. Nevertheless, from the here obtained HRSTEM and EDX data it is evident that Hf_0.11_Al_0.20_B_0.69_ forms a dense oxide scale containing around 30 at% of Al and B as well as 3 at% of Hf, as determined within the known limitations of EDX.

**Fig. 6:**
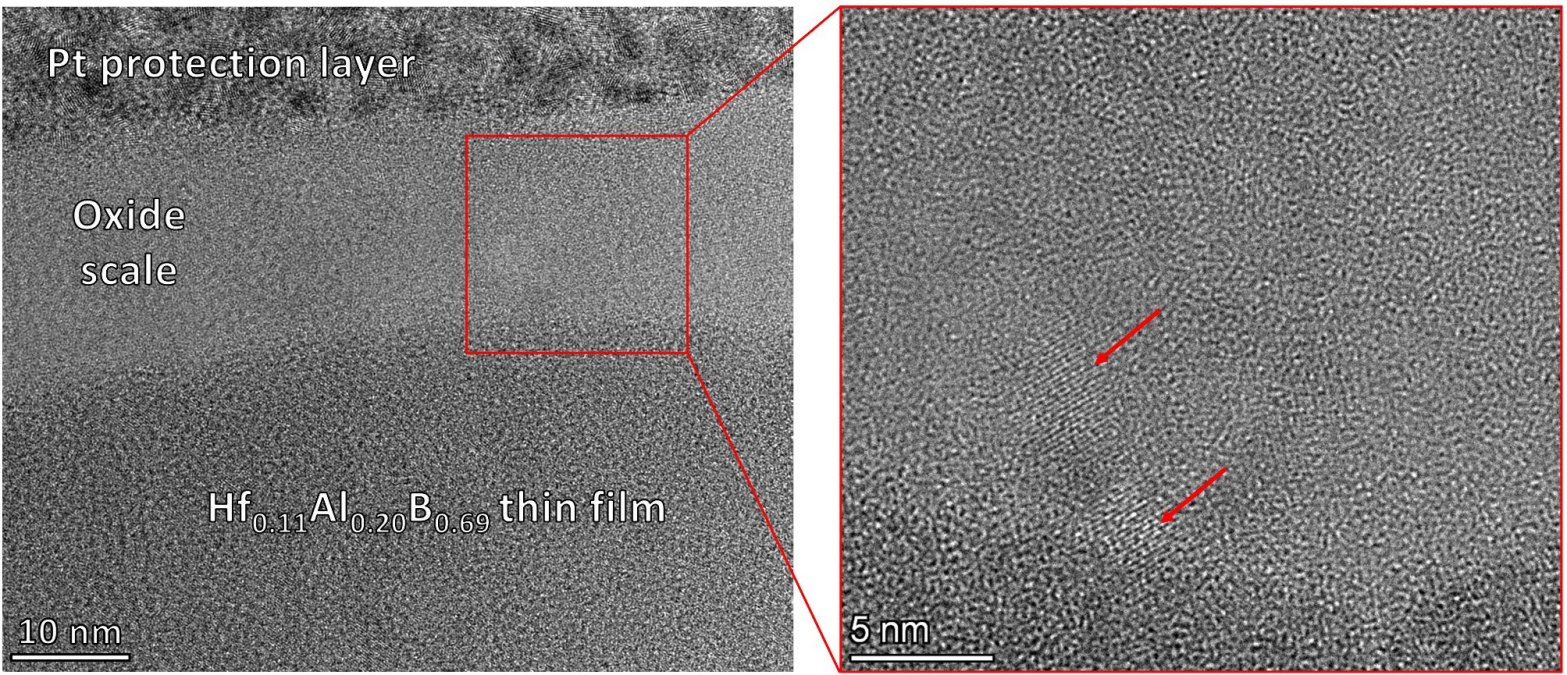
Typical cross-sectional DF TEM image of the oxide scale of Hf_0.11_Al_0.20_B_0.69_. The red arrows mark the nanocrystalline areas.

Based on XRD the oxide forming on Hf_0.11_Al_0.20_B_0.69_ is amorphous. Transmission electron microscopy (TEM) dark field (DF) images of the interface between the oxide scale and film, which are depicted in Fig. 6, also show a mostly amorphous oxide scale. However, nanocrystalline regions, marked with red arrows, are visible preferentially in the vicinity of the interface which may have formed during oxidation or electron beam exposure. The unoxidized film is crystalline and the interface between oxide and film is well defined.

**Fig. 7:**
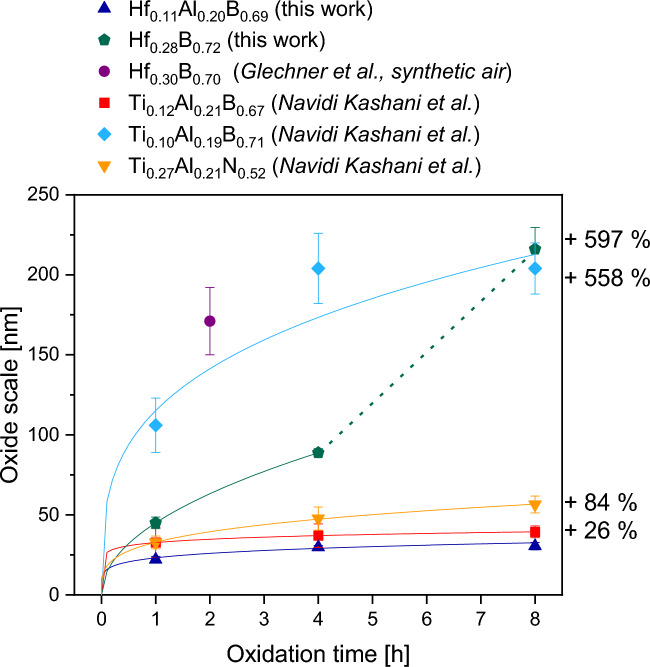
Oxide scale thickness as a function of oxidation time at 700 °C for Hf_0.28_B_0.72_ and Hf_0.11_Al_0.20_B_0.69_ compared to the available literature data from Glechner et al.^24^ and Navidi Kashani et al.^33,34^. The dashed line for Hf_0.28_B_0.72_ is included as a guide for the eye.

To study the oxide growth kinetics, Fig. 7 shows the resulting oxide scale thicknesses of the film determined by STEM imaging on FIB-prepared lamellae as a function of oxidation time at 700 °C. Also, the relevant literature data, namely magnetron sputtered Hf_0.30_B_0.70_^24^, Ti_0.12_Al_0.21_B_0.67_^33^, Ti_0.10_Al_0.19_B_0.71_^33^_,_ and Ti_0.27_Al_0.21_N_0.52_^34^ thin films, are added for comparison. After 1 h of oxidation at 700 °C, Hf_0.28_B_0.72_ exhibits an oxide layer thickness of 45 $\pm$ 4 nm. During further oxidation, this thickness increases significantly from 89 $\pm$ 3 nm after 4 h to 216 $\pm$ 14 nm after 8 h. Oxidation of Hf_0.11_Al_0.20_B_0.69_ at 700 °C after 1 h results in the formation of a 22 $\pm$ 1 nm thick oxide layer, which increases to 30 $\pm$ 1 nm and 31 $\pm$ 2 nm after 4 and 8 h, respectively.

To study the oxidation behavior and the effect of the Al addition on the oxidation kinetics, a power law fit according to^50^ was utilized.

2 $$\Delta x = {K}^{\prime}{\left(\frac{t}{{t}_{0}}\right)}^{n}$$

The oxide scale thickness $\Delta x$ is measured in $\mu m$, ${t}_{0}=1 s$, *t* is the oxidation time measured in seconds, ${K}^{\prime}$ is the pre-exponential factor and $n$ is the time exponent, where exponents of 0.5 and 0.33 would indicate parabolic and cubic oxidation kinetics, respectively. The pre-exponential factor ${K}^{\prime}$ and the time exponent $n$ determined for the films compared in Fig. 7 are listed in Supplementary Table 1.

Initially, the Hf_0.28_B_0.72_ thin film shows a parabolic oxidation behavior with n $=$ 0.49. During that initial state, no oxide peaks occur in XRD diffractograms, indicating the formation of an X-ray amorphous Hf and B containing oxide scale. However, beyond 4 h of oxidation at 700 °C, the oxide scale crystallizes and pore formation in the oxide scale increases leading to the acceleration of oxidation kinetics as indicated by the dashed line to guide the eye and thus a non-passivating behavior. The observation that Hf_0.29_B_0.71_ shows initially slow oxidation kinetics at 700 °C which then increases after a certain time has been reported previously^24^.

In contrast, Hf_0.11_Al_0.20_B_0.69_ shows with n $=$ 0.16 a much slower, sub-cubic oxidation rate. The substantially improved oxidation resistance of Hf_0.11_Al_0.20_B_0.69_ compared to Hf_0.29_B_0.71_ can be rationalized based on the XRD, EDX, HRSTEM and TEM data: firstly, the morphologies of the formed oxides vary significantly: While on Hf_0.29_B_0.71_ the formation of a porous, crystalline oxide scale is observed, oxidation of Hf_0.11_Al_0.20_B_0.69_ results in a dense, amorphous and passivating oxide scale. Secondly, the chemical composition of the oxide varies significantly: while Hf_0.29_B_0.71_ forms a Hf-rich oxide scale containing significant amounts of B, Hf_0.11_Al_0.20_B_0.69_ forms an oxide scale with only small amounts of Hf but high Al and B concentration.

Next, the influence of the transition metal on the oxidation resistance is discussed by comparing Hf_0.09_Al_0.21_B_0.70_ with over-stoichiometric Ti_0.10_Al_0.19_B_0.71_ and stoichiometric Ti_0.12_Al_0.21_B_0.67_^33^. It is evident from Fig. 7 that Ti_0.12_Al_0.21_B_0.67_ and Hf_0.11_Al_0.20_B_0.69_ show a similar oxidation behavior, however, the oxide thickness of Ti_0.12_Al_0.21_B_0.67_ is 26% thicker than for Hf_0.11_Al_0.20_B_0.69_ after 8 h of oxidation. It is noteworthy that the here investigated Hf_0.11_Al_0.20_B_0.69_ is both, over-stoichiometric with a boron-to-metal ratio of 2.2 and superior in oxidation behavior compared to stoichiometric Ti_0.12_Al_0.21_B_0.67_. As pointed out in the introduction the boron-to-metal ratio plays a major role in the oxidation resistance of Ti–Al–B thin films as a comparison with the over-stoichiometric Ti_0.10_Al_0.19_B_0.71_^33^ illustrates. The over-stoichiometric Ti_0.10_Al_0.19_B_0.71_ thin film shows an increase in oxide scale thickness by factor 5.2 resulting in 204 ± 16 nm compared to stoichiometric Ti_0.12_Al_0.21_B_0.67_ after oxidation at 700 °C for 8 h. The reason is the formation of a porous oxide scale, which is enriched in Ti and O as well as depleted in Al and B and is therefore less oxidation resistant. In contrast, the stoichiometric Ti_0.12_Al_0.21_B_0.67_ film forms a dense, Al-rich oxide scale which is highly oxidation resistant^33^ as a passivating scale is formed^34^. The Hf_0.11_Al_0.20_B_0.69_ film shows excellent oxidation resistance even though a significant amount of B is in the Al-rich oxide scale.

Another advantage of Hf_0.11_Al_0.20_B_0.69_ compared to Ti–Al–B films with a similar Al concentration^33^ is the enhancement of mechanical properties. The highly oxidation-resistant Ti_0.12_Al_0.21_B_0.67_ exhibits hardness and elastic modulus values of 19 $\pm$ 1 GPa and 395 $\pm$ 12 GPa, respectively. The elastic modulus is similar to the Hf_0.11_Al_0.20_B_0.69_ film, however, the hardness is significantly reduced compared to 30 $\pm$ 1 GPa of Hf_0.11_Al_0.20_B_0.69_. Higher hardness is observed for the over-stoichiometric Ti_0.10_Al_0.19_B_0.71_ with 24 $\pm$ 1 GPa, but still significantly lower than for Hf_0.11_Al_0.20_B_0.69_. Moreover, the elastic modulus of over-stoichiometric Ti_0.10_Al_0.19_B_0.71_ is only 330 $\pm$ 9 GPa, and the oxidation resistance is much inferior with an oxide scale thickness that is 558% larger than the one of Hf_0.11_Al_0.20_B_0.69_^33^.

Finally, Hf_0.11_Al_0.20_B_0.69_ is compared to the industrial benchmark coating Ti_0.27_Al_0.21_N_0.52_^34^. After oxidation at 700 °C for 8 h, the nitride film exhibits an oxide scale thickness of 57 ± 5 nm corresponding to an 84% increased thickness compared to Hf_0.11_Al_0.20_B_0.69_. This data shows that Hf_0.11_Al_0.20_B_0.69_ exhibits superior combined mechanical and oxidation behavior at 700 °C compared to Ti–Al-based diborides and nitrides, even when the boron-to-metal ratio is over-stoichiometric. The fact that the oxidation resistance of Hf-Al-based diborides, in contrast to Ti–Al-based diborides, is not adversely affected by over-stoichiometric B concentrations results in less stringent requirements concerning the composition distribution obtained within an industrial deposition system.

## Conclusion

Single-phase solid solution Hf_0.29_B_0.71_ and Hf_0.11_Al_0.20_B_0.69_ thin films with a hexagonal crystal structure were synthesized by magnetron sputtering to investigate the phase formation and oxidation behavior of Hf-Al-B thin films for the first time. DFT calculations of the mixing enthalpy of Hf-Al-B predict a metastable solid solution and the DFT-calculated lattice parameters are in very good agreement with the experimentally obtained lattice parameters *a* and *c*.

Hf_0.29_B_0.71_ thin films exhibit a hardness of 38 $\pm$ 2 GPa and an elastic modulus of 541 $\pm$ 19 GPa. The addition of Al, forming Hf_0.11_Al_0.20_B_0.69,_ leads to a reduction in hardness and elastic modulus to 30 $\pm$ 1 GPa and 397 $\pm$ 9 GPa, respectively. Both experimentally determined elastic modulus values are in good agreement with DFT predictions and can be understood by considering the corresponding changes in cohesive energy showing Al concentration induced bond weakening. After isothermal oxidation at 700 °C for 8 h of Hf_0.29_B_0.71_, an oxide scale thickness of 216 $\pm$ 14 nm was obtained. When Hf_0.11_Al_0.20_B_0.69_ was oxidized under the same conditions the oxide scale thickness was 31 $\pm$ 2 nm, which corresponds to an 86% reduction compared to Hf_0.29_B_0.71_. This behavior can be explained by comparing the chemical composition and the morphology of the corresponding oxide scales. While on Hf_0.29_B_0.71_ a porous, O, Hf, and B-containing scale is formed with crystalline HfO_2_ peaks observed by XRD, on Hf_0.11_Al_0.20_B_0.69_ a dense, predominantly amorphous scale containing O, Al, B as well as small amounts of Hf is formed, which reduces the oxidation kinetics significantly by passivation.

While B over-stoichiometry leads to a deterioration in oxidation behavior in Ti–Al-based diborides^31,33^, B-rich Hf_0.11_Al_0.20_B_0.69_ shows sub-cubic oxidation kinetics similar to stoichiometric Ti_0.12_Al_0.21_B_0.67_^33^. As a result, the oxide layer thickness of Ti_0.10_Al_0.19_B_0.71_ is 558% thicker compared to Hf_0.11_Al_0.20_B_0.69_, because Hf_0.11_Al_0.20_B_0.69_ exhibits an oxide scale that is denser and less enriched in the transition metal than the scale of Ti_0.10_Al_0.19_B_0.71_^33^. A comparison of Hf_0.11_Al_0.20_B_0.69_ and Ti_0.12_Al_0.21_B_0.67_^33^ thin films shows that despite the B over-stoichiometry in Hf_0.11_Al_0.20_B_0.69_, Ti_0.12_Al_0.21_B_0.67_ exhibits a 26% thicker oxide scale, indicating that the O diffusion through the Al_2_O_3_ and B_2_O_3_ containing oxide scale is slowed down by the incorporation of approximately 3 at% of Hf even if the B concentration in the Hf_0.11_Al_0.20_B_0.69_ oxide scale seems to be larger than for Ti_0.12_Al_0.21_B_0.67_. Hf_0.11_Al_0.20_B_0.69_ outperforms well-established Ti_0.27_Al_0.21_N_0.52_^33^ regarding oxidation behavior since the oxide scale thickness of the nitride is 84% thicker than on Hf_0.11_Al_0.20_B_0.69_.

This study highlights the untapped application potential of Hf-Al-based diboride thin films for oxidation protection. Very significantly, in comparison to Ti–Al-based diborides, Hf-Al-based diborides have the advantage of forming passivating oxide scales for B-rich thin film compositions, thereby improving processability while exhibiting a high hardness of 30 $\pm$ 1 GPa.

## Methods

### Experimental methods

The Hf_0.28_B_0.72_ and Hf_0.11_Al_0.20_B_0.69_ thin films were synthesized by direct current magnetron sputtering using an in-house built high-vacuum growth system with a base pressure of less than 6 × 10^–4^ Pa at the deposition temperature of 400 °C. Stoichiometric 2-inch HfB_2_ (99.8% purity) and AlB_2_ (99.8% purity) compound targets (Plansee Composite Materials GmbH) were mounted at a 45° angle with respect to the substrate normal as depicted in Supplementary Fig. 1.

For the Hf_0.11_Al_0.20_B_0.69_ deposition, the HfB_2_ and AlB_2_ targets were co-sputtered at a constant power density of 3.9 W/cm^2^ and 7.4 W/cm^2^, respectively. To deposit the reference Hf_0.28_B_0.72_ thin film only the HfB_2_ target was operated at a constant power density of 7.4 W/cm^2^. All films were grown in pure Ar atmosphere (99.9999% purity) at 0.6 Pa and floating substrate bias potential onto 10 × 10 mm^2^ α-Al_2_O_3_ (0001) substrates with a target-to-substrate distance of 10 cm. Substrate rotation was used to ensure a homogenous composition. The deposition time was chosen so both film thicknesses were 1 µm with deposition rates of 13.3 nm/min for Hf_0.11_Al_0.20_B_0.69_ and 11.1 nm/min for Hf_0.28_B_0.72_.

The samples were oxidized in ambient air at 700 °C for exposure times of 1, 4, and 8 h using a GERO tube furnace. The as-deposited samples were placed into an Al_2_O_3_ crucible with an attached Ni/Ni–Cr thermocouple and transferred into the pre-heated furnace at 700 °C and immediately removed from the furnace after the exposure time.

For the structural analysis, a Bruker AXS D8 Discover General Area Detector Diffraction System was used where the Cu K_α_ (λ $=$ 1.5406 Å) X-ray source was set to 40 kV at a current of 40 mA and the incident angle was fixed at 15°. The peaks were fitted with a pseudo-Voigt II function using the TOPAS software V.3. Based on the fitted peak positions, lattice parameters were calculated using the CellCalc software V. 2.10.

For determining the chemical composition, time-of-flight elastic recoil detection analysis (ToF-ERDA) was carried out at the Tandem Accelerator Laboratory of Uppsala University^51^. Recoils were generated using a 36 MeV ^127^I^8+^ primary ion beam and time-energy coincidence spectra were recorded with a detector telescope, equipped with thin carbon foils for ToF measurement and a gas detector for energy discrimination^52^. Further information on the detector telescope and the ToF setup is available in reference^53^. Uncertainties regarding the stopping power values as well as the detection efficiency dominate the measurement uncertainty^54^ and beam straggling and multiple scattering diminish the depth resolution for heavy transition metals like Hf^55^. For Ti-B films it has been reported that ERDA and Rutherford backscattering spectrometry deviate less than 3% and thus coincide very well^23^. Since ERDA is employed as a stand-alone technique in the present work, a maximum total uncertainty of 5% of the deduced values is assumed for the light element B and aliquot fractions thereof for the metals (Hf,Al). The composition was quantified from the surface near region of the depth profile to prevent an influence of multiple scattering on the chemical composition analysis^23,55,56^.

The mechanical properties of as-deposited films were determined by quasistatic nanoindentation in a Hysitron TI-900 TriboIndenter. Indentation modulus as well as hardness were derived according to the method from Oliver and Pharr^57^. A diamond indenter (Poisson's ratio of 0.07, elastic modulus of 1140 GPa) with Berkovich geometry was used. Load-controlled measurements with 6 mN were applied, resulting in maximum contact depths of 60 nm for as-deposited samples, corresponding to < 6% of the film thickness. 50 indents were performed for each sample and the indenter area function was derived from measurements on fused silica. Measured indentation modulus values were converted to the elastic modulus with a Poisson's ratio of 0.154 for HfB_2_, obtained by averaging published data from density functional theory (DFT) calculations^58–61^. For Hf_0.11_Al_0.20_B_0.69_ a Poisson's ratio of 0.131 was used, based on the interpolation between HfB_2_ and the calculated value of 0.118 for AlB_2_^34^.

Scanning transmission electron microscopy (STEM) was performed to study the microstructure of as-deposited and oxidized samples in a dual-beam microscope FEI Helios NanoLab 600 with a Ga^+^ focused ion beam (FIB) and equipped with an EDAX Octane Elect energy dispersive X-ray spectroscopy (EDX) detector. First, the lamellae were prepared with a standard lift-out procedure, and then STEM imaging was carried out at an acceleration voltage of 30 kV and a current of 50 pA.

Transmission electron microscopy (TEM) was utilized to study the microstructure and chemical composition using a Thermo Fisher Scientific Titan Themis 200 G3 equipped with a SuperX detector for EDX mapping. The TEM lamellae were first prepared in the FEI Helios NanoLab 600 as well and were subsequently thinned and polished at 5 kV using a Tescan Lyra FIB/SEM system.

### Computational methods

The lattice constants of relaxed Hf_0.33-*x*_Al_*x*_B_0.67_ with *x* = 0, 0.04, 0.09, 0.17, 0.24, and 0.33 were calculated based on DFT^62,63^ as implemented in the Vienna ab initio simulation package (VASP)^64,65^. The projector augmented-plane-wave method^66,67^ was used with the generalized gradient approximation with PBE^68^ functional described the exchange–correlation effects. The plane-wave cutoff energy was set to 500 eV, and a 7 × 7 x 7 k-point mesh was used. For the calculations, 3 × 3 x 3 supercells of the AlB_2_-type (P6/mmm) were created. The metal sublattice was populated with Hf and Al using the Special Quasi-random Structures method as implemented in^69^. The X-ray diffraction (XRD) patterns from the calculated structures were determined with the VESTA software^70^. The elastic constants were calculated in the form of the full stiffness tensor (C_ij_), from which the Young's modulus was derived with the Voigt-Reuss-Hill scheme^71^. The stiffness tensor was obtained with the strain–stress method^72^.

## Supplementary Information

Supplementary Information.

## Data Availability

The data and samples analyzed in the current study are either included in the article and in the supplementary information or can be obtained from the corresponding author on request.
