# Author Correction: Improved oxidation behavior of Hf_0.11_Al_0.20_B_0.69_ in comparison to Hf_0.28_B_0.72_ magnetron sputtered thin films

**DOI:** 10.1038/s41598-024-75854-8

**Published:** 2024-10-24

**Authors:** Pauline Kümmerl, Sebastian Lellig, Amir Hossein Navidi Kashani, Marcus Hans, Peter J. Pöllmann, Lukas Löfler, Ganesh Kumar Nayak, Damian M. Holzapfel, Szilárd Kolozsvári, Peter Polcik, Peter Schweizer, Daniel Primetzhofer, Johann Michler, Jochen M. Schneider

**Affiliations:** 1 Materials Chemistry, RWTH Aachen University, Kopernikusstr. 10, 52074 Aachen, Germany; 2 Empa, Swiss Federal Laboratories for Materials Science and Technology, Laboratory for Mechanics of Materials and Nanostructures, Feuerwerkerstrasse 39, 3602 Thun, Switzerland; 3 Plansee Composite Materials GmbH, Siebenbürgerstr. 23, 86963 Lechbruck am See, Germany; 4 Department of Physics and Astronomy, Uppsala University, Lägerhyddsvägen 1, 75120 Uppsala, Sweden

Correction to: *Scientific Reports* 10.1038/s41598-024-72134-3, published online 17 September 2024

The original version of this Article contained an error in the Methods section, under the subheading ‘Experimental methods’.

“For determining the chemical composition, time-of-flight elastic recoil detection analysis (ToF-ERDA) was carried out at the Tandem Accelerator Laboratory of Uppsala University [10.1088/1748-0221/17/04/P04011].”

now reads:

“For determining the chemical composition, time-of-flight elastic recoil detection analysis (ToF-ERDA) was carried out at the Tandem Accelerator Laboratory of Uppsala University^51^.

The original Article has been corrected.

