# Supplementary material for: Improved oxidation behavior of Hf0.11Al0.20B0.69 in comparison to Hf0.28B0.72 magnetron sputtered thin films
Source: Sci Rep. 2024 Sep 17;14:21653. doi: 10.1038/s41598-024-72134-3 (PMC11408622; doi:10.1038/s41598-024-72134-3)
Supplement: Supplementary file 1 — Supplementary Information. [file 41598_2024_72134_MOESM1_ESM.pdf]

Supplementary information

Improved oxidation behavior of $Hf_{0.11}Al_{0.20}B_{0.69}$ in comparison to $Hf_{0.28}B_{0.72}$ magnetron sputtered thin films

Pauline Kümmerl[1*], Sebastian Lellig[1,2], Amir Hossein Navidi Kashani[1], Marcus Hans[1], Peter J. Pöllmann[1], Lukas Löfler[1], Ganesh Kumar Nayak[1], Damian M. Holzapfel[1], Szilárd Kolozsvári[3], Peter Polcik[3], Peter Schweizer[2], Daniel Primetzhofer[4], Johann Michler[2] and Jochen M. Schneider[1]

[1] Materials Chemistry, RWTH Aachen University, Kopernikusstr. 10, D-52074 Aachen, Germany

[2]Empa, Swiss Federal Laboratories for Materials Science and Technology, Laboratory for Mechanics of Materials and Nanostructures, Feuerwerkerstrasse 39, CH-3602 Thun, Switzerland

[3]Plansee Composite Materials GmbH, Siebenbürgerstr. 23, D-86963 Lechbruck am See, Germany

[4]Department of Physics and Astronomy, Uppsala University, Lägerhyddsvägen 1, S-75120 Uppsala, Sweden

* Corresponding author; E-mail: kuemmerl@mch.rwth-aachen.de

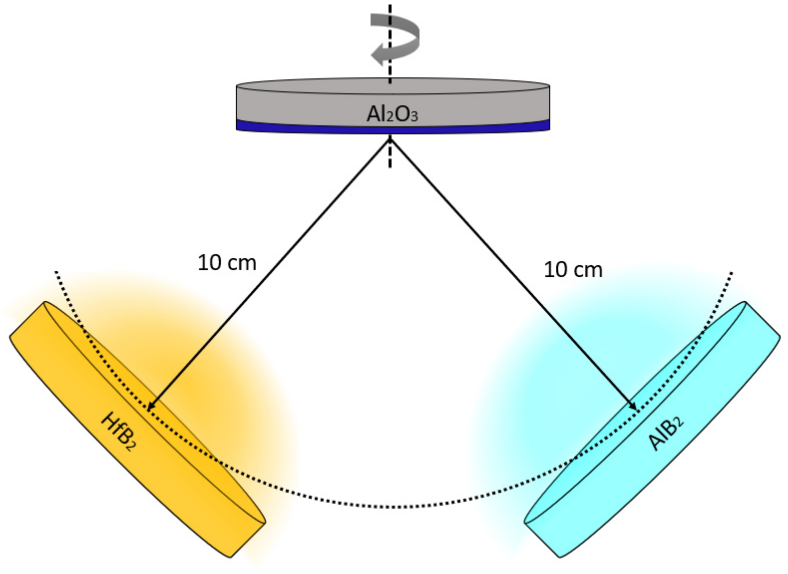

Supplementary Figure 1: Deposition set up in the in-house built high-vacuum growth system.

Supplementary Table 1: Fitting parameters K' (pre-exponential factor) and n (time exponent) for the films depicted in Figure 7. For $Hf_{0.28}B_{0.72}$ the fitting parameters are only valid for up to 4 h of oxidation, afterwards the kinetics increase.

	K'	n	Reference
$Hf_{0.11}Al_{0.20}B_{0.69}$	0.006	0.16	
$Hf_{0.28}B_{0.72}$	0.001	0.49	
$Ti_{0.12}Al_{0.21}B_{0.67}$	0.015	0.10	[33]
$Ti_{0.10}Al_{0.19}B_{0.71}$	0.013	0.35	[34]
$Ti_{0.27}Al_{0.21}N_{0.52}$	0.004	0.26	[34]
